# When expert identity helps and hurts: a double-edged sword effect of expert identity on adaptive performance among flexibly recruited professionals

**DOI:** 10.3389/fpsyg.2025.1705081

**Published:** 2025-12-03

**Authors:** Dongheng Han, Xun Cui

**Affiliations:** Business School, Nankai University, Tianjin, China

**Keywords:** expert identity, adaptive performance, role stress, work engagement, flexibly recruited professionals, conservation of resources theory (COR)

## Abstract

In the context of the rapid growth of the sharing and platform economy, flexible recruitment has become an important way for organizations to access critical knowledge and technical support, enhancing agility and innovation. The adaptive performance of flexibly recruited professionals reflects their ability to adapt to and perform in part-time service organizations, directly affecting the value they create. However, research on its mechanisms remains limited. Drawing on Conservation of Resources (COR) theory, this study views expert identity as a key psychological resource. It can promote work engagement, triggering a resource gain spiral that enhances adaptive performance. Conversely, it may also induce role stress, leading to a resource loss spiral that hinders adaptive performance. Organizational support from the primary organization functions as an important moderating role, strengthening the positive pathway and buffering the negative one. A two-wave survey of high-skilled professionals with flexible recruitment experience yielded 244 valid responses. Regression and structural equation modeling (SEM) analyses supported the hypotheses. This study integrates COR theory into the flexible recruitment context, uncovering dual gain-loss mechanisms of expert identity and enriching the antecedent research on adaptive performance. Practically, it highlights the need for organizations to value such talent and foster positive relationships with their primary organizations to maximize the contributions that flexibly recruited professionals create.

## 1 Introduction

In the era of the rapidly growing sharing economy and platform economy, organizations have increasingly adopted diverse and flexible approaches to acquiring high-level talent (Kalleberg, 2003; Lin and Joe, 2012; Barley et al., 2017). In fact, flexible recruitment is not entirely new. Earlier organizational practices already included forms such as external consultants, part-time researchers, and independent professionals (Kalleberg, 2000; Hoque and Kirkpatrick, 2003; McKeown and Pichault, 2021; Sulbout et al., 2022). However, the development of new digital platforms and cooperative mechanisms has significantly expanded the flexibility of collaboration between firms and professionals, thus greatly accelerating the growth of flexible recruitment. In the Chinese context, this phenomenon is particularly distinctive. Beyond market demand, national and local governments have also introduced policies encouraging firms and high-level talent to engage in project collaboration, scientific research, training, and consulting through flexible arrangements (Ying, 2018; Liu, 2021). Against this backdrop, this study focuses on a specific group of nonstandard employees: flexibly recruited professionals (FRPs). These individuals typically hold core positions in their primary organizations, possess extensive expertise and experience, and use their non-working hours to provide professional services to secondary or host organizations (hereafter referred to as part-time service organizations) in the form of part-time work, collaboration, or consulting. As a critical source of knowledge and technical capacity for part-time service organizations, FRPs play an increasingly important role in organizational development.

Nevertheless, the extent to which FRPs can truly create value for part-time service organizations does not solely depend on their expertise and experience. Because they operate in cross-organizational contexts and assume multiple roles, they must demonstrate the ability to adapt quickly and effectively to different tasks and environments, which is precisely adaptive performance. Adaptive performance refers to employees’ ability to adjust flexibly and perform effectively when confronted with uncertain situations, unexpected events, and novel tasks (Pulakos et al., 2000). For FRPs, adaptive performance is a key determinant of the degree to which their value is realized in part-time service organizations.

Existing research on nonstandard employment has primarily focused on gig workers, freelancers, temporary agency workers, and multiple jobholders (Lopes and Chambel, 2014; Ashford et al., 2018; Campion et al., 2020; McKeown and Pichault, 2021). Much of this work examines low-skilled or temporary laborers, exploring their motivations for choosing nonstandard employment (e.g., income supplementation, autonomy) (Muzzolon et al., 2015; Lopes and Chambel, 2017; Sobral et al., 2021) and the potential spillover effects on their primary jobs (Sessions et al., 2021; Sessions et al., 2023; Liu et al., 2025). A small stream of research has turned attention to multiple jobholders in white-collar or core positions, investigating how secondary employment experiences feed back into their primary job performance (Caza et al., 2022a; Robertson et al., 2024). However, FRPs, as a group of highly skilled, cross-organizational nonstandard professionals, have received limited scholarly attention. While the antecedents of adaptive performance have been examined to some extent, most studies have focused on full-time employees’ adaptability within organizational boundaries (e.g., across departments or job roles) (Jundt et al., 2015; Park and Park, 2019), with insufficient consideration of adaptive capabilities in cross-organizational contexts. Furthermore, FRPs face particularly complex identity challenges. On one hand, identity research has largely emphasized organizational and professional identities among formal employees within a single organization (Hekman et al., 2009; Brown and Coupland, 2015; Caza et al., 2018). On the other hand, studies of nonstandard employment have tended to explore either temporary agency workers’ organizational identification with host organizations or gig workers’ professional identity, reflecting how they perceive their career development or occupational belonging (George and Chattopadhyay, 2005; Vallas and Christin, 2018; Caza et al., 2022b; Yang et al., 2022; Akkermans et al., 2025). Compared with these groups, FRPs possess dual attributes of formal and nonstandard employment, and the formation and implications of their identities remain underexplored. Building on this gap, this study investigates the mechanisms by which FRPs’ expert identity influences their adaptive performance in part-time service organizations.

To reveal this mechanism, we employ Conservation of Resources (COR) theory (Hobfoll, 1989) as the guiding theoretical framework. COR theory posits that individuals strive to acquire, retain, and protect valued resources, whereby the accumulation of resources may trigger a “resource gain spiral,” while resource loss can initiate a “resource loss spiral” (Halbesleben et al., 2014). Building on this framework, we propose two mediating mechanisms: work engagement and role stress, which represent the resource gain path and resource depletion path of expert identity, respectively. On the one hand, expert identity, as an important psychological resource, enhances individuals’ sense of self-worth and meaningfulness at work, thereby energizing them to dedicate greater focus and effort, which is manifested as higher work engagement (May et al., 2004; Karanika-Murray et al., 2015). Prior research suggests that when individuals strongly identify with a role or group, they are more willing to devote additional effort to tasks associated with that role (Ashforth et al., 2008; Mao et al., 2023). Accordingly, expert identity may foster higher levels of work engagement, which in turn enhances FRPs’ adaptive performance in part-time service organizations. On the other hand, strong expert identity may also generate stress. Overemphasizing one’s expert role may lead individuals to experience role conflict and ambiguity when navigating across organizations, or role overload stemming from multiple expectations (Elovainio and Kivimäki, 2001; Sun et al., 2016). Such role stress depletes psychological resources and may undermine their adaptability in part-time service organizations. Thus, expert identity may negatively affect adaptive performance via the resource depletion path of role stress.

Furthermore, consistent with COR theory, external resources play a critical role in shaping how individuals manage resource gain and loss spirals (Hobfoll, 2001; Hobfoll et al., 2018). In the context of flexible recruitment, organizational support from the primary organization emerges as one of the most vital external resources. First, such support enhances FRPs’ sense of resource security, alleviating concerns about potential resource loss during cross-organizational collaboration (Rhoades and Eisenberger, 2002; Al Nahyan et al., 2024). Second, it provides socioemotional incentives, whereby employees who feel valued and supported are more motivated to exert extra effort across multiple roles (Eisenberger et al., 2020). Finally, higher levels of organizational support from the primary organization reduce FRPs’ concerns about dual identities, enabling them to engage more fully in the tasks of part-time service organizations while maintaining their primary job responsibilities, thus improving resource utilization efficiency. Consequently, organizational support from the primary organization may not only amplify the positive pathway through which expert identity promotes adaptive performance via work engagement, but also buffer the negative pathway through which expert identity undermines adaptive performance via role stress.

To sum up, the current study develops a dual-path model of FRPs’ expert identity and adaptive performance based on COR theory. We analyze the mediating roles of work engagement and role stress in linking expert identity to adaptive performance, and examine the moderating role of primary organizational support. This study makes several theoretical contributions. First, by conceptualizing and clarifying FRPs, we extend research on nonstandard employment beyond low-skilled or multiple jobholders to encompass highly skilled, cross-organizational professionals, thereby enriching the theoretical scope of nonstandard employment research. Second, by highlighting the double-edged nature of expert identity, which operates through the mechanisms of resource gain and resource depletion (Hobfoll, 1989; Hobfoll, 2001), this study advances identity research and extends COR theory into nonstandard employment relationships. Finally, by incorporating organizational support from the primary organization as a boundary condition, we show that external resources can simultaneously amplify and buffer the effects of identity, thereby refining COR theory’s core propositions and offering new insights into how FRPs mobilize and allocate resources across dual organizational contexts.

## 2 Theory and research hypothesis

### 2.1 The mediating role of work engagement

For flexibly recruited professionals (FRPs), the absence of formal employment in part-time service organizations deprives them of institutional guarantees and organizational belonging. Consequently, they rely more heavily on their psychological resources to sustain proactive work behaviors. In this context, expert identity, defined as the self-affirmation of professional competence and role value (Crane, 2012; Kang and Kim, 2022), serves as a critical psychological resource enabling FRPs to display adaptive behaviors in their work tasks within part-time service organizations. The conservation of resources (COR) theory posits that individuals strive to acquire, preserve, and enhance valued resources and convert these resources into energy for action when confronted with situational challenges (Hobfoll, 1989; Hobfoll, 2014). Extant research indicates that when individuals develop a strong identification with a particular role, they are more inclined to devote additional effort to tasks associated with that role in order to sustain and validate their identity (Ashforth et al., 2008). Thus, for FRPs, expert identity constitutes an investable resource that enhances self-efficacy and role meaning, thereby motivating them to engage more deeply in their work tasks. Work engagement is conceptualized as a positive psychological state characterized by vigor, dedication, and absorption at work (Schaufeli et al., 2006). Prior studies have identified antecedents such as psychological meaningfulness, psychological safety, organizational commitment and the alignment between role-related values and resource provision (Rich et al., 2010; Woods and Sofat, 2013; Karanika-Murray et al., 2015; Guo et al., 2022; Siyal, 2023). Building on these findings, we argue that expert identity can serve as another important psychological resource that promotes work engagement, particularly for FRPs working in part-time service organizations. Within part-time service organizations, expert identity provides the psychological momentum of “resource gain,” encouraging FRPs to invest greater time and energy in work tasks to demonstrate and reinforce their expert identity. Therefore, we propose:

*Hypothesis 1a:* Expert identity positively influences the work engagement of FRPs in part-time service organizations.

The value of FRPs in part-time service organizations is reflected not only in the knowledge and skills they contribute but also in their capacity to adjust rapidly and perform effectively in dynamic cross-organizational tasks. Adaptive performance is defined as employees’ ability to flexibly adjust and engage in continuous learning when faced with novel tasks, uncertain environments, or unexpected events (Pulakos et al., 2000; Griffin et al., 2010; Park and Park, 2021). Given that FRPs are required to navigate organizational boundaries and balance multiple roles, adaptive performance is a decisive factor shaping their role value and collaborative potential. Prior studies have identified multiple antecedents of adaptive performance, including learning orientation and cognitive flexibility, emotional intelligence, and job autonomy and social support provided by organizations (Jundt et al., 2015; Park and Park, 2019; Balti and Karoui Zouaoui, 2023; Ouellette et al., 2024). These findings suggest that adaptive performance arises from the joint influence of individual psychological traits and contextual resources. Building on this perspective, we contend that work engagement functions as a critical antecedent of adaptive performance. Engaged employees are more likely to demonstrate learning capacity and proactive behaviors in complex environments, thereby enhancing their task performance and adaptability (Christian et al., 2011). Within the framework of COR theory, work engagement reflects the mobilization of resources into behavioral advantages, enabling individuals to draw on cognitive and emotional capacities to learn, adapt, and problem-solve in uncertain contexts (Halbesleben et al., 2014; Hobfoll et al., 2018). Hence, when FRPs maintain high levels of work engagement in part-time service organizations, they are more likely to grasp task demands, mobilize cross-organizational resources, and display higher levels of adaptive performance. Therefore, we propose:

*Hypothesis 1b:* Work engagement positively influences the adaptive performance of FRPs in part-time service organizations.

In summary, expert identity operates as a pivotal psychological resource that promotes adaptive performance by enhancing work engagement. By strengthening the sense of role meaning, expert identity motivates FRPs to devote greater time and effort to their work tasks in part-time service organizations. High levels of work engagement, in turn, enable them to mobilize and accumulate resources more effectively, thereby enhancing their ability to manage uncertainty and role challenges. COR theory suggests that such processes of accumulation and investment give rise to a “resource gain spiral,” which generates additional resources and leads to higher performance outcomes (Hobfoll et al., 2018). On this basis, we propose:

*Hypothesis 1c:* Work engagement mediates the relationship between expert identity and the adaptive performance of FRPs in part-time service organizations.

### 2.2 The mediating role of role stress

Although expert identity is generally considered a positive psychological resource, in the cross-organizational context of flexibly recruited professionals (FRPs), it can also become a source of stress. FRPs are required to fulfill core responsibilities in their primary organizations while simultaneously meeting the high expectations of part-time service organizations regarding their expert roles, which creates additional psychological burdens during role transitions. Prior studies suggest that while identity reinforcement can enhance individuals’ sense of meaning, in contexts where multiple roles coexist, it may also intensify tension and strain (Elovainio and Kivimäki, 2001; Horton et al., 2014). According to COR theory, when individuals devote substantial resources to maintain and demonstrate a salient identity, yet face external role demands that exceed their capacity, they are likely to enter a spiral of resource depletion (Hobfoll, 2001; Halbesleben et al., 2014). For FRPs, a strong sense of expert identity may motivate them to respond to the expectations of part-time service organizations, but it simultaneously increases the likelihood of experiencing inter-role conflict and overload, thereby raising their overall level of role stress. Therefore, we propose:

*Hypothesis 2a:* Expert identity positively influences the role stress of FRPs.

In the high-intensity and dynamic work environments of part-time service organizations, FRPs are expected to contribute their professional expertise as well as switch flexibly across organizational boundaries and role identities. Prolonged exposure to elevated role stress intensifies the depletion of their personal resources; furthermore, it imposes substantive constraints on their capacity to adapt to organizational change. Empirical evidence has shown that role stress is a critical negative antecedent of performance and adaptive behaviors, as it may cause cognitive overload, emotional exhaustion, and motivational decline, thereby reducing individuals’ ability to respond proactively in uncertain environments (de Ruyter et al., 2001; Duxbury et al., 2018; Tang and Vandenberghe, 2021; Zhao and Jiang, 2022). For FRPs, the need to simultaneously meet the demands of their primary organizations and the high expectations of part-time service organizations for their expert roles further amplifies the complexity of role stress. As stress accumulates, FRPs are forced to continually expend their limited psychological resources to cope, which suppresses their learning, adaptability, and innovation in dynamic contexts. Employees under conditions of resource scarcity and high pressure are more likely to adopt conservative strategies to address immediate demands, which comes at the expense of proactive engagement with new tasks and environments (Hobfoll, 2001; Halbesleben et al., 2014). From this perspective, role stress represents a typical resource-depleting mechanism that steadily erodes individuals’ energy and adaptability, thereby constraining their performance in dynamic organizational contexts. Over the long term, FRPs who have to continuously navigate conflicting expectations and identity-related pressures will find it increasingly difficult to sustain high levels of adaptive performance. Thus, we propose:

*Hypothesis 2b:* Role stress negatively influences the adaptive performance of FRPs in part-time service organizations.

To summarize, in the cross-organizational employment context of FRPs, strong expert identity may bring additional psychological burdens that increase role stress. This role stress faced by FRPs not only depletes psychological and cognitive resources but also inhibits proactive learning and adaptability, ultimately impairing adaptive performance in part-time service organizations. In this process, role stress mediates the relationship between expert identity and adaptive performance. The COR theory suggests that when individuals invest heavily in maintaining an important identity, such as expert identity, yet encounter excessive demands across multiple organizational settings, their resources may be consumed faster than they can be replenished, leading to a spiral of resource loss (Hobfoll et al., 2018). In this sense, role stress functions as a resource-depleting mechanism that weakens individuals’ ability to cope with complex environments and diminishes their performance. Based on above analysis, we propose the following:

*Hypothesis 2c:* Role stress mediates the relationship between expert identity and the adaptive performance of FRPs in part-time service organizations.

### 2.3 The moderating role of organizational support from primary organization

Given that flexibly recruited professionals (FRPs) in part-time service organizations lack the institutional guarantees and sense of belonging typically linked to formal employment, they place particular emphasis on support from their primary organizations to sustain resource security and strengthen their psychological foundation for cross-organizational work (De Cuyper and De Witte, 2007). This study defines such support as organizational support from the primary organization, which refers to FRPs’ perception that their primary organization recognizes and supports their value and contributions both emotionally and resource-wise. This concept represents a contextualized adaptation of perceived organizational support (POS) to the FRP setting (Eisenberger et al., 1986). Prior research shows that organizational support enhances employees’ sense of security and being valued, which in turn stimulates work motivation (Gillet et al., 2013; Kurtessis et al., 2017), and also encourages extra-role effort through social exchange mechanisms (Thompson et al., 2020). Therefore, organizational support from the primary organization functions to help FRPs mitigate uncertainty during identity transitions across organizations, while also providing essential psychological and emotional resources for their work in part-time service organizations. From the perspective of COR theory, this support can be regarded as a key external resource. External resources not only offer direct protection but also complement internal resources (Hobfoll et al., 2018). In this study, organizational support from the primary organization may amplify the resource-gain effects of expert identity while buffering its resource-depleting processes, thereby acting as a crucial contextual factor within the dual-pathway mechanism.

On the one hand, in the relationship between expert identity and work engagement, organizational support from the primary organization may serve as an enhancing factor. Research suggests that employees who perceive a high level of organizational support tend to experience stronger psychological safety and a greater sense of responsibility, making them more willing to invest additional effort and emotion in their work (Tucker et al., 2008; Eisenberger et al., 2020). For FRPs, when their primary organizations provide sustained recognition and support, such external resources strengthen their sense of resource security and facilitate the translation of expert identity into high levels of work engagement in part-time service organizations. Put differently, organizational support from the primary organization signals affirmation of FRPs’ engagement in flexible recruitment, and it also reduces the opportunity costs they might face when committing resources to part-time service organizations. According to COR theory, external resources often activate the potential of internal resources, enabling individuals to transform identity-based motivation into concrete work behaviors and thereby fostering a “resource gain spiral” in which continuous resource accumulation promotes positive performance (Halbesleben et al., 2014; Hobfoll et al., 2018). Conversely, when organizational support from the primary organization is lacking, FRPs may withhold effort in part-time service organizations despite strong expert identity, as they fear resource depletion. Thus, we propose:

*Hypothesis 3:* Organizational support from the primary organization moderates the positive relationship between expert identity and work engagement of FRPs, such that this relationship is stronger when organizational support from the primary organization is higher rather than lower.

On the other hand, in the relationship between expert identity and role stress, organizational support from the primary organization may serve as a buffering factor. Although expert identity can reinforce FRPs’ sense of responsibility and role meaning, it may simultaneously impose additional identity burdens that increase role stress (Haslam et al., 2005). However, when FRPs perceive high levels of support from their primary organizations, this external resource can mitigate stress in multiple ways. Emotional support reduces feelings of isolation and threat associated with heightened identity salience, while instrumental support provides additional assurance and resources to help manage multiple demands, thus enabling FRPs to better balance dual roles (Jawahar et al., 2007). COR theory suggests that external resources not only help individuals cope with immediate stressors but also prevent further resource loss, thereby avoiding a “resource loss spiral” (Halbesleben et al., 2014). Accordingly, when organizational support from the primary organization is high, the positive relationship between expert identity and role stress should weaken significantly. In contrast, under conditions of low support, the strengthening of expert identity is more likely to transform into a resource-depleting process, elevating role stress. Therefore, we propose:

*Hypothesis 4:* Organizational support from the primary organization moderates the positive relationship between expert identity and role stress of FRPs, such that this relationship will be weaker when organizational support from the primary organization is higher rather than lower.

**Figure 1:**
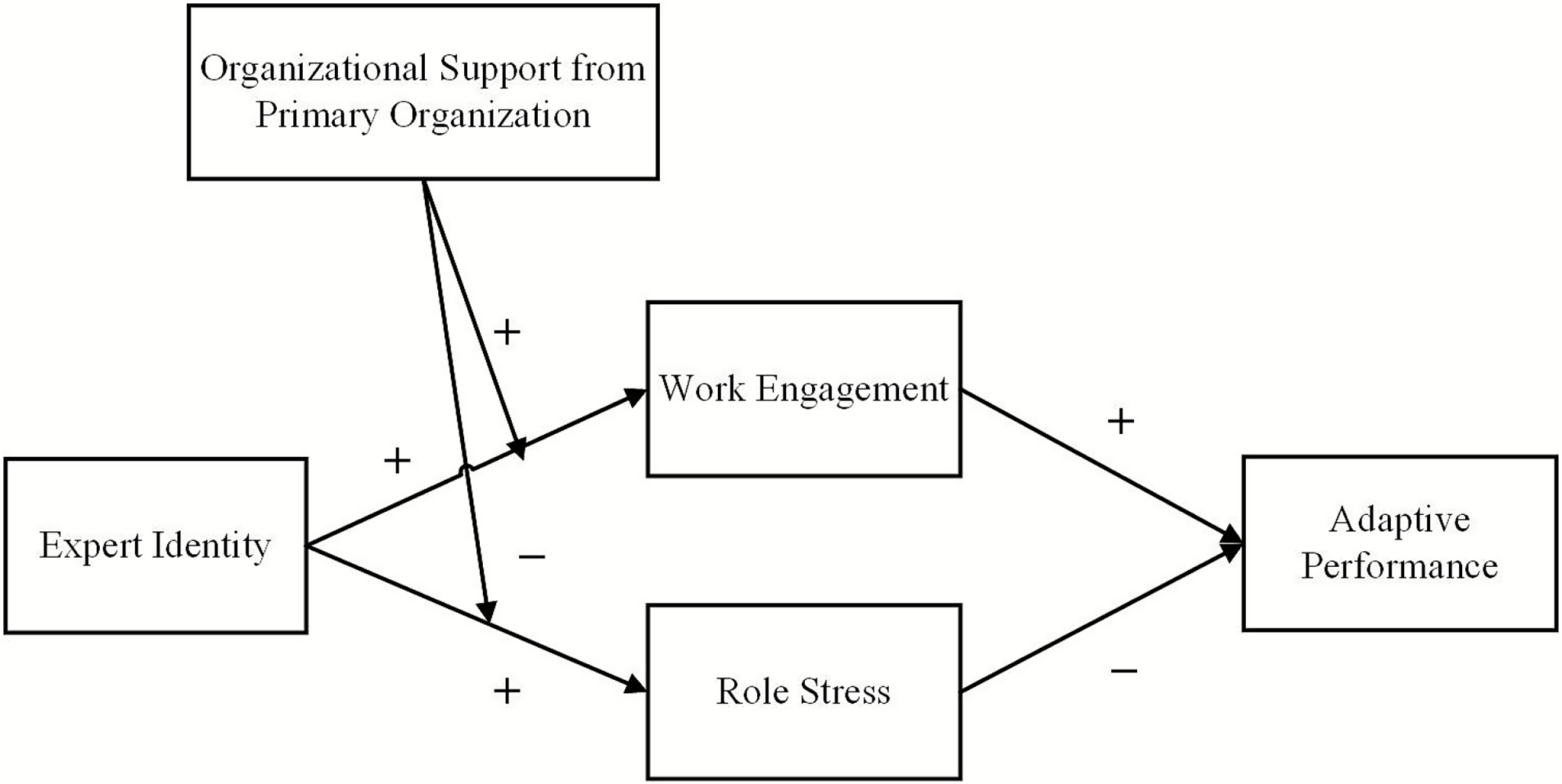
Theoretical model.

In summary, this study argues that expert identity promotes the adaptive performance of FRPs through work engagement (H1a–H1c), while it undermines such performance through role stress (H2a–H2c). At the same time, organizational support from the primary organization moderates the relationship between expert identity and work engagement (H3) as well as the relationship between expert identity and role stress (H4). This suggests that organizational support from the primary organization influences both how identity translates into engagement or stress and how these mediating mechanisms affect adaptive performance. In other words, this study proposes a moderated mediation model (see Figure 1). Specifically, high levels of organizational support from the primary organization strengthen the translation of expert identity into work engagement, thereby amplifying its positive indirect effect on adaptive performance. Conversely, under low levels of organizational support, this indirect effect may be weaker. Correspondingly, high levels of organizational support buffer the translation of expert identity into role stress, thereby reducing its negative indirect effect on adaptive performance. Under low levels of organizational support, however, this negative indirect effect may become more pronounced. Drawing on the above theoretical analysis, we propose:

*Hypothesis 5:* Organizational support from the primary organization moderates the mediated relationship between expert identity and adaptive performance of FRPs via work engagement, such that this relationship is stronger when organizational support from the primary organization is higher rather than lower.

*Hypothesis 6:* Organizational support from the primary organization moderates the mediated relationship between expert identity and adaptive performance of FRPs via role stress, such that this relationship is weaker when organizational support from the primary organization is higher rather than lower.

## 3 Methodology

### 3.1 Participants and procedure

Given the limited size and dispersed distribution of the population of flexibly recruited professionals (FRPs), it is challenging to obtain a large-scale sample through conventional sampling methods. To effectively reach this rare group and enhance external validity, we employed a snowball sampling strategy (Brislin, 1970), which is commonly used in studies of scarce or hard-to-reach populations, such as research on high-level talent mobility and specialized occupational groups.

In the initial stage, the researchers contacted several university faculty members, employees in core technical positions, and senior executives through personal networks of family and friends. These individuals were interviewed to confirm whether they had engaged in flexible recruitment activities, such as project-based collaboration, consulting, or training, with organizations other than their primary employers. Eligible participants were then informed of the survey’s scope, research purpose, and confidentiality assurances, and were invited to complete an online questionnaire distributed via email or social media. They were also encouraged to forward the questionnaire to colleagues or friends who met the inclusion criteria, using internal organizational contact lists, personal social networks, or professional associations, thereby expanding the sample pool.

Data were collected in two waves. Time 1 measured participants’ demographic information, expert identity, and organizational support from the primary organizations. One month later (Time 2), the questionnaire was administered to the same participants, assessing work engagement, role stress, and adaptive performance. Several procedural controls were implemented to ensure data quality. First, the two-wave design and matching of responses via the last four digits of participants’ mobile phone numbers helped mitigate common method bias. Second, screening questions and reasonable time limits for survey completion were used to identify and exclude careless or hasty responses. Third, the survey introduction emphasized the academic purpose of the research and the anonymity of responses, addressed participants’ concerns, and clarified that there were no right or wrong answers, thus enhancing response authenticity.

At Time 1, 302 questionnaires were returned, of which 291 were valid. At Time 2 (1 month after Time 1), questionnaires were distributed to these 291 participants, yielding 261 returns and 257 valid responses. Matching across the two waves resulted in 244 valid responses, for an effective response rate of 80.79%. Among these respondents, 60.2% were male and 39.8% were female; 3.7% were under 25 years old, 23.8% were aged 26–35, 40.1% were aged 36–45, 21.7% were aged 46–55, and 9.8% were over 56. Participants with a master’s or doctoral degree accounted for 64.8% of the sample. In terms of primary job, university faculty and researchers in academic institutions represented 58.6%, while technical R&D staff and managers in enterprises accounted for 34.8%. Regarding years of flexible recruitment experience, 55.3% had 1 year or less, 24.6% had 2 to 5 years, 14.3% had 5 to 10 years, and 5.7% had more than 10 years.

### 3.2 Measurements

All variables in this study were measured using well-established scales that have been widely applied in domestic and international research and demonstrated good reliability and validity. The scales were adapted to fit the research context where necessary. For original English version instruments, we employed a rigorous translation-back translation procedure (Brislin, 1970). After drafting the initial items, two experts in nonstandard employment research and three participants with flexible recruitment experience evaluated the logical consistency and clarity of the items. Revisions were made based on their feedback. Except for control variables, all variables were measured using a five-point Likert scale (1 = “strongly disagree,” 5 = “strongly agree”).

#### 3.2.1 Expert identity

Expert identity was measured using the six-item scale developed by Hekman et al. (2009). Sample items include: “When someone praises the group of flexibly recruited professionals, I also feel proud” and “I am happy when others regard me as a flexibly recruited professional.” This scale has been adapted in prior research to measure role-based identities across different contexts, making it suitable for capturing FRPs’ expert identity. The Cronbach’s *α* for this scale was 0.895.

#### 3.2.2 Role stress

Role stress was measured using the scale developed by Peterson et al. (1995), which includes three dimensions: role conflict (3 items), role ambiguity (5 items), and role overload (5 items). Sample items include: “When working in the part-time service organization, I often have to deal with a number of conflicting demands” (role conflict), “I am certain about how much authority I have” (role ambiguity), and “I feel that the amount of work I am expected to do is too great” (role overload). The Cronbach’s *α* for this scale was 0.970.

#### 3.2.3 Work engagement

Work engagement was assessed using the Utrecht Work Engagement Scale (UWES) developed by Schaufeli et al. (2006), which has been validated in the Chinese context. We retained the three original dimensions (vigor, dedication and absorption), each with three items, and adapted the wording to the flexible recruitment context. Sample items include: “When working in the part-time service organization, I feel bursting with energy” (vigor), “I am enthusiastic about my work in the part-time service organization” (dedication), and “I am immersed in my work for the part-time service organization” (absorption). The Cronbach’s α for this scale was 0.967.

#### 3.2.4 Adaptive performance

Adaptive performance was measured using the Chinese version of the scale developed by Tao and Wang (2006), adapted from Pulakos et al. (2000). It comprises four dimensions: handling emergencies and crisis (7 items), cultural and interpersonal adaptability (8 items), continuous learning (6 items), and solving problems creatively (4 items). Sample items include: “I can think clearly and prioritize effectively when handling urgent problems” (handling emergencies and crisis), “I can adjust my behavior to fit in with other cultures and customs” (cultural and interpersonal adaptability), “I actively learn new techniques and methods related to my job” (continuous learning), and “I can develop creative solutions to complex problems” (solving problems creatively). The Cronbach’s α for this scale was 0.987.

#### 3.2.5 Organizational support from primary organization

Organizational support from the primary organization was measured using the simplified version of the Survey of Perceived Organizational Support (SPOS) developed by Eisenberger et al. (1986). The scale contains six items, such as “When I have a problem, my primary organization will try to help me” and “My primary organization really cares about my well-being.” The Cronbach’s α for this scale was 0.975.

#### 3.2.6 Control variables

We controlled for gender, age, education level, and years of experience in flexible recruitment because previous studies showed that they might influence key variables (Chambel et al., 2015; Sessions et al., 2021; Sessions et al., 2023).

## 4 Results

To ensure the validity and robustness of the results, we first conducted Harman’s single-factor test using SPSS 26.0 to assess potential common method bias. Next, we employed confirmatory factor analysis (CFA) with AMOS 24.0 to examine the measurement model and evaluate construct validity. We then computed descriptive statistics and correlations among the study variables using SPSS 26.0. Finally, we tested the hypothesized mediation and moderation effects with the PROCESS macro for SPSS, and applied bootstrapping procedures to generate bias-corrected confidence intervals.

### 4.1 Confirmatory factor analysis

We conducted confirmatory factor analysis (CFA) using AMOS 24.0 to examine the discriminant validity of the five core variables in the study: expert identity, work engagement, role stress, adaptive performance, and organizational support from the primary organization. As shown in Table 1, the hypothesized five-factor model provided the best fit to the data (*χ*^2^/df = 2.284, CFI = 0.885, TLI = 0.881, SRMR = 0.046, RMSEA = 0.073) compared to all alternative models. These results support the distinctiveness of the five constructs.

**Table 1:** Confirmatory factor analysis result.

Model	*χ* ^2^	*df*	*χ*^2^/*df*	RMSEA	SRMR	CFI	TLI
Five-factor model	3750.034	1,642	2.284	0.073	0.046	0.885	0.881
Four-factor model	6030.884	1,646	3.664	0.105	0.141	0.762	0.752
Three-factor model	6901.199	1,649	4.185	0.114	0.158	0.714	0.704
Two-factor model	8879.82	1,651	5.378	0.134	0.176	0.607	0.593
One-factor model	14657.936	1710	8.572	0.177	SRMR	0.397	0.376

*N* = 244. Five-factor model: expert identity; adaptive performance; work engagement; role stress; organizational support from primary organization. Four-factor model: expert identity; adaptive performance; work engagement + role stress; organizational support from primary organization. Three-factor model: expert identity + adaptive performance; work engagement + role stress; organizational support from primary organization. Two-factor model: expert identity + adaptive performance; work engagement + role stress + organizational support from primary organization. One-factor model: expert identity + adaptive performance + work engagement + role stress + organizational support from primary organization.

To further address the issue of common method bias, we conducted Harman’s single-factor test using SPSS 24.0. The results showed that the largest variance explained by a single factor was 39.946%, below the critical threshold of 40% (Podsakoff et al., 2003), indicating that common method bias was unlikely to be a serious concern in this study.

### 4.2 Descriptive statistics

Table 2 presents the means, standard deviations, and correlations of the variables in this study. As shown, expert identity was significantly and positively correlated with work engagement (*r* = 0.285, *p* < 0.01) and role stress (*r* = 0.289, *p* < 0.01). Work engagement was positively correlated with adaptive performance (*r* = 0.423, *p* < 0.01), whereas role stress was negatively correlated with adaptive performance (*r* = −0.393, *p* < 0.01). These correlations are consistent with our theoretical expectations and provide preliminary support for the proposed hypotheses.

**Table 2:** Means, standard deviations, and correlations.

Variables	1	2	3	4	5	6	7	8	9
1. Gender	1								
2. Age	−0.101	1							
3. Education	−0.007	0.028	1						
4. Years of flexible recruitment experience	−0.058	0.452^**^	−0.151^*^	1					
5. Expert identity	−0.128^*^	0.074	0.027	0.028	1				
6. Work engagement	−0.082	0.018	0.032	0.131^*^	0.285^**^	1			
7. Role stress	0.006	0.003	−0.011	−0.163^*^	0.289^**^	−0.291^**^	1		
8. Adaptive performance	−0.033	−0.013	−0.194^**^	0.197^**^	0.006	0.423^**^	−0.393^**^	1	
9. Organizational support from primary organization	−0.027	−0.114	0.011	−0.067	0.311^**^	0.173^**^	0.133^*^	−0.055	1
Mean	1.398	3.102	2.988	1.705	4.106	3.72	3.676	4.033	3.911
Standard deviation	0.49	0.995	0.918	0.918	0.733	1.066	1.022	0.664	0.893

*N* = 244. ^*^p < 0.05; ^**^p < 0.01.

### 4.3 Hypothesis testing

To test the hypothesized mediation and moderation effects, we employed the PROCESS macro in SPSS. Specifically, Model 4 was used to examine the mediation effects, and Model 7 was used for the moderated mediation analyses. Bias-corrected bootstrapping with 5,000 resamples was applied to estimate 95% confidence intervals (CI) for indirect effects, following recommended best practices.

The results for the mediation model are presented in Table 3. Expert identity significantly and positively predicted work engagement (*β* = 0.379, *p* < 0.001), supporting Hypothesis 1a. Work engagement, in turn, positively predicted adaptive performance (*β* = 0.316, *p* < 0.001), supporting Hypothesis 1b. As shown in Table 4, the indirect effect of expert identity on adaptive performance via work engagement was 0.120, with a 95% confidence interval (CI) of [0.057, 0.196], indicating a significant positive indirect effect. Therefore, Hypothesis 1c was supported.

**Table 3:** Results of mediating effect test.

Variables	Work engagement	Role stress		Adaptive performance		
	Model 1	Model 2	Model 3	Model 4	Model 5	Model 6
Expert identity	0.379^***^	0.403^***^	0.006	−0.114^*^	0.119^*^	−0.016
Work engagement				0.316^***^		0.249^***^
Role stress					−0.280^***^	−0.180^***^
*R* ^2^	0.069	0.084	0	0.234	0.170	0.294
∆*R*^2^	0.065		−0.004	0.227	0.163	0.285
*F*	17.996^***^	22.093^***^	0.009	36.754^***^	24.675^***^	33.278^***^

*N* = 244. ^*^p < 0.05; ^**^p < 0.01; ^***^p < 0.001.

**Table 4:** Results of indirect effects (*N* = 244).

Indirect effects	Estimate	*SE*	95% CI	
			LLCI	ULCI
Expert identity→Work engagement→Adaptive performance	0.120	0.036	0.057	0.196
Expert identity→ Role stress→Adaptive performance	−0.113	0.026	−0.165	−0.063
Total indirect effect	0.022	0.041	−0.055	0.109

Similarly, expert identity significantly and positively predicted role stress (*β* = 0.403, *p* < 0.001), supporting Hypothesis 2a. Role stress negatively predicted adaptive performance (*β* = −0.280, *p* < 0.001), supporting Hypothesis 2b. The indirect effect of expert identity on adaptive performance via role stress was −0.113, with a 95% CI of [−0.165, −0.063], indicating a significant negative indirect effect. Therefore, Hypothesis 2c was supported.

To examine the moderating role of organizational support from the primary organization in the relationship between expert identity and the mediators, we employed PROCESS macro (Model 7), consistent with our theoretical framework. All analyses used bias-corrected bootstrapping with 5,000 resamples to generate robust 95% confidence intervals (CIs) for the conditional effects.

**Figure 2:**
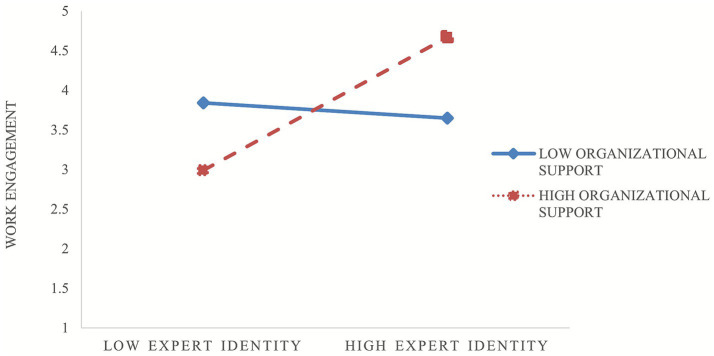
The moderating role of organizational support from the primary organization in the relationship between expert identity and work engagement.

The results in Table 5 indicated that expert identity had a significant positive effect on work engagement among flexibly recruited professionals (*β* = 0.405, *p* < 0.01). Moreover, the interaction term between organizational support from the primary organization and expert identity was positively and significantly associated with work engagement (*β* = 0.099, *p* < 0.01), suggesting that organizational support from the primary organization strengthened the positive relationship between expert identity and work engagement. Specifically, the higher the level of organizational support, the stronger the positive effect of expert identity on work engagement (see Figure 2). Therefore, Hypothesis 3 was supported.

**Table 5:** Analysis of moderating effects on work engagement.

Variables	Work engagement			
	*β*	*SE*	*t*	*p*
Intercept	2.264	0.538	4.212	0.000**
Expert identity	0.405	0.091	4.450	0.000**
Organizational support from the primary organization	−0.094	0.080	−1.167	0.245
Expert identity * Organizational support from the primary organization	0.099	0.034	2.886	0.004**
*R* ^2^	0.089			
Adjusted *R*^2^	0.069			
F	*F* = 4.651, *p* = 0.000			

^*^p < 0.05; ^**^p < 0.01.

**Figure 3:**
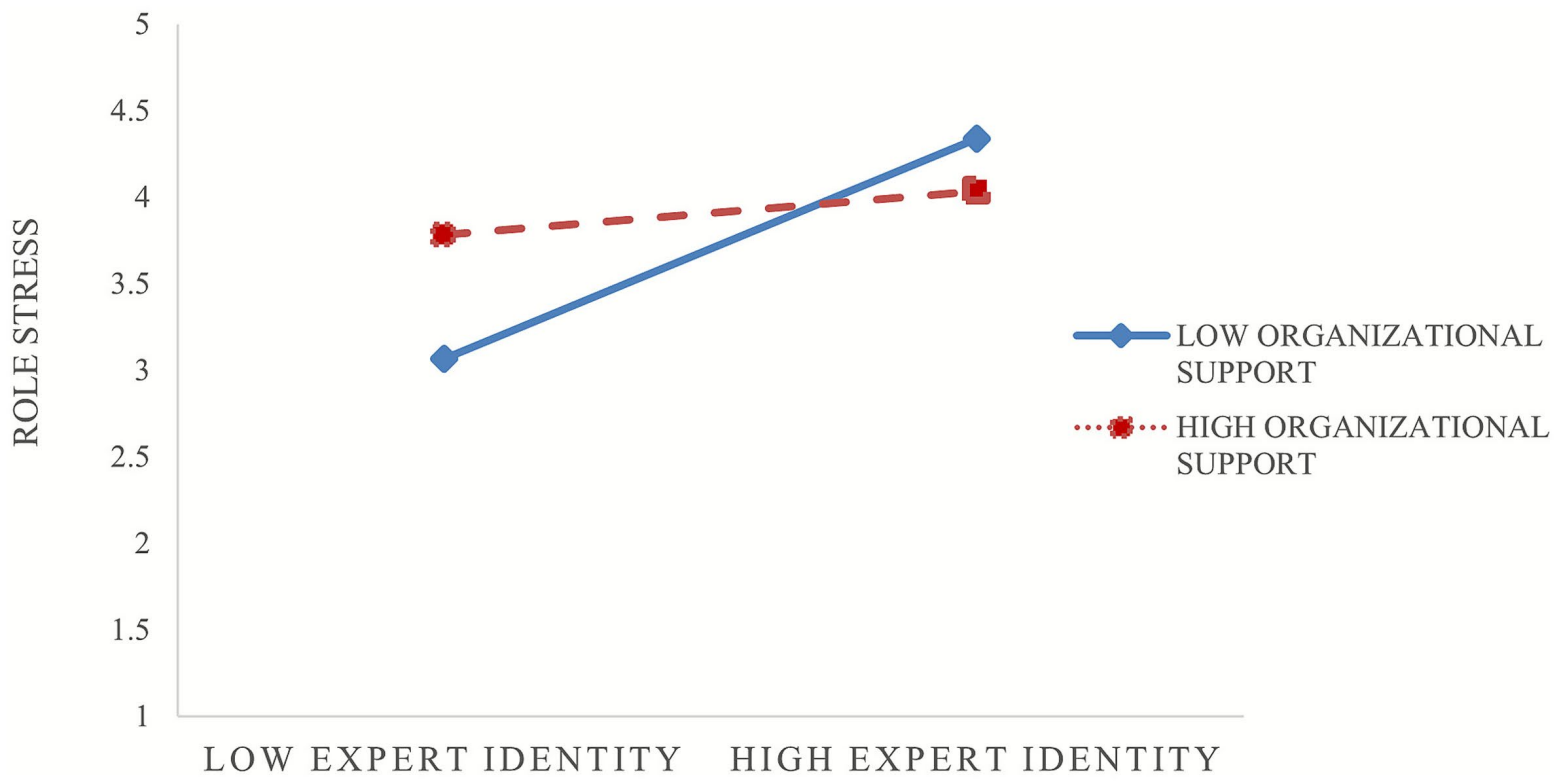
The moderating role of organizational support from the primary organization in the relationship between expert identity and role stress.

Similarly, the results in Table 6 showed that expert identity exerted a significant positive effect on role stress (*β* = 1.377, *p* < 0.01), while the interaction between organizational support from the primary organization and expert identity had a significant negative effect on role stress (*β* = −0.255, *p* < 0.05). This indicates that organizational support from the primary organization weakened the positive relationship between expert identity and role stress, such that the effect was weaker at higher levels of organizational support (see Figure 3). Hypothesis 4 was therefore supported.

**Table 6:** Analysis of moderating effects on role stress.

Variables	Role stress			
	*β*	*SE*	*t*	*p*
Intercept	−2.254	1.708	−1.320	0.188
Expert identity	1.377	0.395	3.491	0.000^**^
Organizational support from the primary organization	1.149	0.430	2.675	0.008^**^
Expert identity * Organizational support from the primary organization	−0.255	0.099	−2.575	0.011^*^
*R* ^2^	0.117			
Adjusted *R*^2^	0.089			
F	*F* = 4.484, *p* = 0.000			

*p < 0.05; ^**^p < 0.01.

We further tested the moderated mediation effects. As shown in Table 7, when organizational support from the primary organization was at a low level, the indirect effect of expert identity on adaptive performance through work engagement was −0.014, with a 95% CI of [−0.082, 0.063], which included zero. At the medium level of organizational support, the indirect effect was 0.109, 95% CI [0.052, 0.179]; and at the high level, the indirect effect was 0.232, 95% CI [0.144, 0.336]. The index of moderated mediation was 0.138, with a standard error of 0.031 and a 95% CI of [0.079, 0.202]. These results indicate that the positive indirect effect of expert identity on adaptive performance through work engagement was stronger when organizational support from the primary organization was higher, supporting Hypothesis 5.

**Table 7:** Results of moderated mediating effect test.

Adaptive performance	Work engagement			Role stress		
	Effect	*SE*	95% CI [LLCI, ULCI]	Effect	*SE*	95% CI [LLCI, ULCI]
Low	−0.014	0.036	[−0.082, 0.063]	−0.171	0.041	[−0.257, −0.099]
Medium	0.109	0.032	[0.052, 0.179]	−0.107	0.027	[−0.160, −0.053]
High	0.232	0.047	[0.144, 0.336]	−0.043	0.036	[−0.108, 0.035]
Index of moderated mediation	0.138	0.031	[0.079, 0.202]	0.071	0.031	[0.018, 0.139]

Similarly, as shown in Table 7 when organizational support from the primary organization was low, the indirect effect of expert identity on adaptive performance via role stress was −0.171, 95% CI [−0.257, −0.099]; at the medium level, the indirect effect was −0.107, 95% CI [−0.160, −0.053]; and at the high level, the indirect effect was −0.043, 95% CI [−0.108, 0.035], which included zero. The index of moderated mediation was 0.071, with a standard error of 0.031 and a 95% CI of [0.018, 0.139]. These findings suggest that higher levels of organizational support from the primary organization weakened the negative indirect effect of expert identity on adaptive performance via role stress. Thus, Hypothesis 6 was supported.

## 5 Discussion

### 5.1 Theoretical implications

First, this study enriches the literature on nonstandard high-skilled workers by identifying flexibly recruited professionals (FRPs) as a unique group operating across organizational boundaries. Rather than focusing solely on traditional forms of nonstandard employment such as gig workers, temporary agency workers or freelancers (Hoque and Kirkpatrick, 2003; Pichault and McKeown, 2019; Caza et al., 2022b; Sulbout et al., 2022), this study demonstrates that FRPs’ adaptive performance in interorganizational collaboration constitutes a critical mechanism through which their value is realized. By introducing adaptive performance into this context, the study deepens theoretical understanding of how high-skilled nonstandard employees respond to complex task demands and contribute to organizational outcomes beyond their primary workplace.

Second, this study reveals the double-edged sword mechanism through which expert identity influences adaptive performance, providing theoretical insights into the dynamics of FRPs. In contrast to the traditional view that treats identity as an unconditionally beneficial psychological resource (Stets and Cast, 2007; Ashforth et al., 2008), this study adopts the perspective of Conservation of Resources (COR) theory to show how expert identity can operate through two functionally distinct mediating paths. Specifically, expert identity enhances work meaning and self-worth, thereby driving greater work engagement, facilitating resource accumulation, and strengthening adaptive performance. Simultaneously, the high centrality of expert identity within FRPs’ self-concept makes them more susceptible to role stress at the intersection of dual organizational boundaries, which induces strain and resource depletion, thereby undermining adaptive performance. These findings not only advance identity research by moving beyond the simple dichotomy of positive versus negative effects but also enrich COR theory by illustrating the dual effects of psychological resources. In doing so, the study provides a novel explanatory framework for understanding the link between identity and adaptive performance.

Third, by introducing organizational support from the primary organization as a key moderating factor, this study sheds light on the crucial role of external resources in shaping the relationship between expert identity and adaptive performance in interorganizational contexts. Previous COR-based research has typically focused on internal organizational resources, such as leader support or team climate (Halbesleben et al., 2014; Hobfoll et al., 2018), with limited attention to how resources are sourced and configured across multiple organizational settings. This study demonstrates that support from the primary organization not only amplifies the positive behavioral pathway triggered by expert identity but also buffers the negative pathway associated with role stress, thereby regulating the dual mechanisms of resource dynamics. Theoretically, this contributes to a more nuanced understanding of the asymmetric effects of external resources and reveals the complexity of resource flows and allocation under dual organizational structures. These insights provide a foundation for future research on cross-organizational resource co-management, talent-sharing mechanisms, and boundary management between organizations.

### 5.2 Practical implications

This study provides several managerial implications for organizations seeking to leverage the value of flexibly recruited professionals (FRPs) in the context of flexible employment. First, part-time service organizations should attach great importance to the adaptive performance of FRPs. FRPs are not only contributors of knowledge and technical expertise but also critical actors who can rapidly adjust and independently respond to task challenges in dynamic environments. To effectively harness the positive impact of expert identity, part-time service organizations may enhance FRPs’ sense of professional value and work meaningfulness by clarifying role expectations, assigning meaningful project tasks, and fostering an open and inclusive organizational climate. In doing so, they can increase FRPs’ work engagement. At the same time, organizations should remain alert to the role conflicts and pressures that may arise from heightened expert identity. On the one hand, they may mitigate resource depletion caused by frequent role switching across organizations by setting reasonable task boundaries and reducing role ambiguity. On the other hand, coordination mechanisms such as “flexible collaboration platforms” or “project coordinators” can be introduced to help FRPs address potential conflicts between their primary and part-time responsibilities, thereby alleviating the negative impact of role stress. Through systematic support, part-time service organizations can more effectively promote FRPs’ adaptive performance and achieve synergies between knowledge acquisition and organizational development.

Second, for primary organizations, flexible recruitment should not be regarded as a threat of talent loss but rather as an opportunity for knowledge spillover and the expansion of talent influence. The findings of this study indicate that perceived organizational support from the primary organization not only enhances FRPs’ sense of resource security and psychological contract but also exerts an important moderating effect during their part-time work process. Specifically, it strengthens the positive pathway through work engagement and buffers the negative pathway through role stress. Therefore, primary organizations should strategically cultivate a supportive organizational climate. This may include granting FRPs flexibility in human resource policies, advocating the openness and positive value of external service in organizational culture, and building cross-organizational platforms for exchange, such as industry alliances or joint research platforms, to establish cooperative relationships with part-time service organizations. Such initiatives serve to enhance FRPs’ resource coordination capacity under dual roles while also strengthening the reputation and influence of primary organizations within their industries. Furthermore, policymakers and platform builders can also play a guiding role by promoting the establishment of a more systematic and standardized framework for flexible recruitment, thereby enabling resource sharing on a broader scale and providing stronger institutional guarantees for FRPs to realize their multiple values.

### 5.3 Limitations and future research

The current study has some limitations that may be addressed by future studies. First, although we employed a multi-wave data collection design, all variables were measured through self-reports. Despite conducting a series of statistical tests to rule out the significant risk of common method bias, concerns about the robustness of the conclusions cannot be fully eliminated (Podsakoff et al., 2003). Future research could incorporate performance evaluations provided by project leaders or colleagues in part-time service organizations, or employ methods such as experience sampling method (ESM) and experimental designs to further validate the dynamic relationships and causal pathways among the variables.

Second, most of the FRPs in our sample were affiliated with universities or research institutes, and only a small proportion came from enterprises. This concentration of respondents within similar institutional environments may have contributed to the clarity of the factor structure and the strength of the observed relationships. This sample composition therefore represents another methodological limitation. Future research may replicate the model using FRPs whose primary organization is based in enterprises or across a wider variety of organizational settings to examine whether the findings remain consistent in more diverse institutional contexts.

Third, although this study draws on Conservation of Resources (COR) theory to propose that expert identity strengthens both resource gains through work engagement and resource depletion through role stress, other potential mediating mechanisms (e.g., psychological empowerment, boundary management strategies) were not included in the model. Future research could broaden the mediating pathways to enrich the explanatory power of the theoretical framework. With regard to moderating mechanisms, this study only examined the role of organizational support from primary organizations. Future research could further investigate contextual factors such as the organizational climate of part-time service organizations or inter-organizational coordination mechanisms, thereby deepening our understanding of resource interactions and boundary conditions in multi-organizational settings.

Fourth, the empirical data were collected in mainland China, where the development of flexible recruitment policies has been strongly shaped by institutional support from local governments. In particular, the strong cultural emphasis on responsibility, work stability, and identity may intensify both the resource-gain pathway (via work engagement) and the resource-depletion pathway (via role stress). It remains unclear whether the proposed dual-path model would operate similarly in Western contexts, where independent professionals or knowledge-based freelancers may not possess formal organizational identities and where a group comparable to FRPs may not exist in the same form. Thus, the cross-cultural generalizability of the findings remains to be tested. Future research could validate the proposed model in other countries or institutional contexts to enhance the external validity and cross-cultural applicability of the theoretical framework.

## 6 Conclusion

Drawing on Conservation of Resources (COR) theory, this study develops a dual-path moderated mediation model to explain how flexibly recruited professionals’ (FRPs) expert identity shapes their adaptive performance in part-time service organizations. The results demonstrate that expert identity contributes to adaptive performance through two distinct resource-related pathways: it enhances work engagement, thereby promoting resource gain, while simultaneously increasing role stress, which leads to resource depletion. In addition, organizational support from the primary organization plays a crucial role in shaping these processes by amplifying the positive pathway through work engagement and mitigating the negative pathway through role stress. Overall, the study advances understanding of how expert identity functions in dual-organizational contexts and highlights the importance of resource dynamics in explaining FRPs’ adaptive performance.

## Data Availability

The original contributions presented in the study are included in the article/Supplementary material, further inquiries can be directed to the corresponding author.
