# Supplementary material for: When expert identity helps and hurts: a double-edged sword effect of expert identity on adaptive performance among flexibly recruited professionals
Source: Front Psychol. 2025 Dec 3;16:1705081. doi: 10.3389/fpsyg.2025.1705081 (PMC12709602; doi:10.3389/fpsyg.2025.1705081)
Supplement: Supplementary file 1 [file Data_Sheet_1.pdf]

Supplementary Material

1 The complete questionnaire

Wave 1

Expert Identity (6 items)

Source: Hekman, D.R., Bigley, G.A., Steensma, H.K., and Hereford, J.F. (2009). Combined effects of organizational and professional identification on the reciprocity dynamic for professional employees Acad. Manage. J. 52(3), 506-526. doi: 10.5465/amj.2009.41330897.

1. When someone praises the group of flexibly recruited professionals, I also feel proud.
2. When someone criticizes the group of flexibly recruited professionals, I feel that I am being criticized as well.
3. When I talk about flexibly recruited professionals, I usually use “we” rather than “they.”
4. I am happy when others regard me as a flexibly recruited professional.
5. Successes such as new theories or new technologies, including publications or approved patents, feel like my own successes.
6. If there is negative media coverage about flexibly recruited professionals, I also take it as a reminder and warning for myself.

Organizational Support From the Primary Organization (6 items)

Source: Eisenberger, R., Huntington, R., Hutchison, S., and Sowa, D. (1986). Perceived organizational support. J. Appl. Psychol. 71(3), 500-507.

1. My primary organization takes my suggestions into consideration.
2. My primary organization looks out for my best interests.
3. My primary organization respects my goals and values.
4. My primary organization helps me when I have difficulties.
5. If I need assistance, my primary organization is willing to provide it.
6. My primary organization shows great concern for my well-being.

Wave 2

Work Engagement (9 items)

Source: Schaufeli, W.B., Bakker, A.B., and Salanova, M. (2006). The measurement of work engagement with a short questionnaire - A cross-national study. Educ. Psychol. Meas. 66(4), 701-716. doi: 10.1177/0013164405282471.

1. I feel mentally vigorous when working in the part-time service organization.
2. I feel full of energy when working in the part-time service organization.
3. I am enthusiastic about my work in the part-time service organization.

4. My work in the part-time service organization inspires me.
5. When I get up in the morning, I look forward to working in the part-time service organization.
6. I feel happy when I am immersed in work in the part-time service organization.
7. I am proud of the work I do in the part-time service organization.
8. I am absorbed in my work in the part-time service organization.
9. I devote my full attention to my work during the time I work in the part-time service organization.

Role Stress (13 items)

Source: Peterson, M.F., Smith, P.B., Akande, A., Ayestaran, S., Bochner, S., Callan, V., et al. (1995). Role conflict, ambiguity, and overload: A 21-nation study. Acad. Manage. J. 38(2), 429-452. doi: 10.2307/256687.

1. When working in the part-time service organization, I often have to deal with many conflicting work demands at the same time.
2. I often receive completely opposing work requests from different people in the part-time service organization.
3. I have to adopt different strategies according to different situations in the part-time service organization.
4. My work arrangements are clear, well-defined, and organized in the part-time service organization.
5. I know exactly what the part-time service organization expects of me.
6. I am very clear about the scope of my responsibilities in the part-time service organization.
7. I clearly understand the level of responsibility I am expected to take on in the part-time service organization.
8. My division of responsibilities in the part-time service organization is quite clear.
9. I wish the part-time service organization would reduce my workload.
10. I feel that my work pressure is too high in the part-time service organization.
11. I feel that I am taking on too much responsibility in the part-time service organization.
12. My workload is so heavy that it affects my ability to maintain work quality in the part-time service organization.
13. My workload is excessive and makes it difficult for me to ensure the quality of my work in the part-time service organization.

Adaptive Performance (25 items)

Source: Tao, Q., and Wang, Z. (2006). The construct of adaptive performance in management training settings. J. of Psychol. Sci. 03, 614-614+579. doi: 10.16719/j.cnki.1671-6981.2006.03.022.

1. I am able to work smoothly with people who have different personalities in the part-time service organization.
2. I am able to adjust my behavior when necessary in the part-time service organization.
3. I take the initiative to understand the atmosphere and needs of other departments in the part-time service organization.
4. I am able to understand the values, climate, and development direction of the part-time service organization.

5. I can integrate myself into different values and customs within the part-time service organization.
6. I continuously adjust my behavior to adapt to other cultures and customs within the part-time service organization.
7. I can easily understand the meaning of behaviors in other cultural contexts and adjust myself accordingly in the part-time service organization.
8. I am able to maintain good relationships with people from different cultural backgrounds in the part-time service organization.
9. I can anticipate possible emergencies and propose several alternative solutions in the part-time service organization.
10. I can handle urgent issues in a clear and well-structured manner in the part-time service organization.
11. I can stay objective and calm when dealing with urgent issues in the part-time service organization.
12. I can take step-by-step measures to solve problems in the part-time service organization.
13. I can remain calm even when under high work pressure in the part-time service organization.
14. I can control my emotions when responding to urgent problems in the part-time service organization.
15. I can stay calm even when work is very tightly scheduled in the part-time service organization.
16. I actively learn new technologies and methods related to my work in the part-time service organization.
17. I can apply newly learned technologies or methods to my actual work in the part-time service organization.
18. I take necessary steps to keep my knowledge and skills up to date.
19. I can quickly master new knowledge and new skills.
20. I can quickly adapt to work procedures and tasks that I have not encountered before.
21. I can compensate for deficiencies that arise in my work through appropriate actions.
22. I can propose innovative ways to solve complex problems in the part-time service organization.
23. I can find solutions to problems through reverse thinking.
24. I can identify solutions to problems based on seemingly irrelevant information.
25. I can find solutions to problems that others have not noticed in the part-time service organization.
